# Consensus-Based Recommendations for Advance Directives of People with Parkinson’s Disease in Regard to Typical Complications by German Movement Disorder Specialists

**DOI:** 10.3390/jcm9020449

**Published:** 2020-02-06

**Authors:** Martin Klietz, Johanna M. Berndt, Florian Wegner, Nils Schneider, Günter U. Höglinger, Carsten Eggers, Stephanie Stiel

**Affiliations:** 1 Department of Neurology, Hannover Medical School, Carl-Neuberg-Straße 1, 30625 Hannover, Germany; Johanna.M.Berndt@stud.mh-hannover.de (J.M.B.); Wegner.Florian@mh-hannover.de (F.W.); Hoeglinger.Guenter@mh-hannover.de (G.U.H.); 2 Institute for General Practice, Hannover Medical School, Carl-Neuberg-Straße 1, 30625 Hannover, Germany; Schneider.Nils@mh-hannover.de (N.S.); Stiel.Stephanie@mh-hannover.de (S.S.); 3 Department of Neurology, University of Marburg, Baldingerstraße, 35037 Marburg, Germany; carsten.eggers@uk-gm.de

**Keywords:** Parkinson’s disease, end of life care, decision-making, Delphi technique, consensus, advance directive, advance care planning

## Abstract

A huge proportion of people with Parkinson’s disease (PwP) in Germany have written an advance directive (AD). However, the content of these forms in regard to specific Parkinson’s disease (PD)-related complications is rather low. There is an urgent need to specify ADs of PwP and consequently to improve decision-making concerning end-of-life aspects for affected patients. Evidence- and consensus-based PD-specific recommendations for ADs might help to close this gap. A Delphi study with two online Delphi rounds was initiated. Initial recommendations were built on findings from previous studies and derived from evidence-based literature. Consensus on recommendations was defined as ≥80% concordance regarding clarity of formulated aspects and relevance for clinical practice. A total of 22 experts (15.2% response rate) predominantly from the workgroup ‘neuro-palliative care’ in Germany performed two Delphi rounds. Consensus was achieved for 14 of 24 initially presented recommendations. Recommendations relating to dopaminergic therapy as well as to non-oral therapy options were considered important by the expert panel. The recommendations should be taken into account when developing and giving advice on ADs for PwP. Health professionals should be trained in counselling ADs of PwP and in integrating these recommendations in ADs during the disease course of PD.

## 1. Introduction

Parkinson’s disease (PD) is a frequent neurodegenerative disease with about 420,000 cases in Germany according to current German health data [1]. After a period of good treatment response, People with PD (PwP) experience several motor and non-motor complications with progressive loss of autonomy, finally leading to care dependence [2,3,4]. PwP suffer from an increased morbidity, mortality and high disease related burden in the course of the movement disorder [3,5,6]. In spite of this, the implementation of palliative care interventions in advanced PD is low [3,7]. Advance care planning (ACP) is a concept that encompasses an early discussion of the patient’s wishes and the resulting therapy as well as the naming of a health care proxy [8]. Since ACP supports adults in understanding and sharing their values, life goals and preferences regarding future medical care [9], it is associated with increased patient satisfaction and quality of life, greater use of palliative care and hospice services and lower rates of unwanted hospitalizations [10,11]. Although ACP planning is gaining international significance for palliative care, in Germany it has so far only been implemented in oncology and to patients in institutional care facilities. While ACP is only offered to a small group of patients in Germany, many of the patients with chronic illnesses have written an advance directive (AD) [3]. AD creation should take place early in the course of a neurodegenerative disease, when the patient is able to make their own choices and can still anticipate the consequences [12].

Therefore, a comprehensive AD is a very important and meaningful tool to ensure the patient’s will in the end-of-life care. Furthermore, the AD is a legally binding document that comes into force when the patient is no longer able to express their own will. To be legally valid in clinical situations, the content of an AD has to be highly specific [13]. In Germany, ADs and determination of a health care proxy are two different legal documents. For this reason, not all patients with AD have named a health care proxy.

In a prior study of our research group we found that a huge proportion (69.7%) of PwP in advanced stages has written an AD. Nevertheless, the content of these ADs is rather unspecific in regard to typical Parkinson’s disease (PD)-related complications such as dopaminergic therapies in an end-of-life setting, non-oral advanced therapies and swallowing disorders [13]. This may be due to a very low rate of neurological consultations for AD creation [13]. There is an urgent need to specify ADs of PwP in order to consequently improve decision-making for affected patients. For example, advance care planning has already been established to improve end-of life care of ALS-patients [14].

For these purposes this study pursuits multiple aims. The first aim of the study is to create recommendations that directly address the treating physicians and neurologists and will serve as guidelines for the temporal and content-related discussion of PD-specific ADs. Second, the study targets the development of consensus-based recommendations that address PD-specific complications in end-of-life care [13]. These recommendations could be added on demand to an already existing AD. However, this study does not aim to create a complete and independent AD for PwP. Results of this study can contribute neurological expertise to frame ACP in PD [15].

## 2. Experimental Section

### 2.1. Ethics Approval

The prior PD patient AD study [8] was approved by the ethics committee of Hannover Medical School (No. 3123-2016 and Amendment 2018). For the presented expert survey, no additional ethics approval was necessary; no additional patient data were included.

### 2.2. Study Design

Taking the current literature and findings from our previous study into account, recommendations addressing specific PD-related complications were developed in a multi professional and interdisciplinary expert group between January and April 2019.

**Figure 1:**
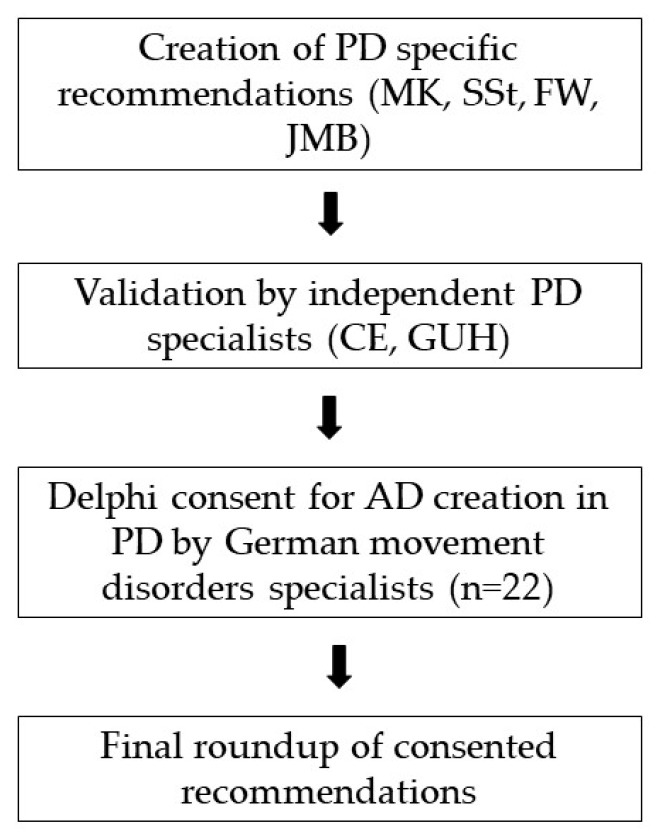
Study design. Abbreviations: PD Parkinson’s disease; AD advance directive.

The developed recommendations were pre-tested by two neurologists and subsequently evaluated in a Germany-wide Delphi process between May and August 2019 (Figure 1). Experts indicated their level of agreement or disagreement by certain answer options. All expert opinions were collected over a number of anonymized Delphi rounds [16] until consensus on the presented statements was reached [17].

### 2.3. Study Population

Experts for this Delphi study were defined as individuals with professional activity in clinical neurology and/or palliative care with special expertise in movement disorders and/or end-of-life care (see Table 1). For data collection, an email distribution list of a German neuro-palliative care group and focus groups of the “Deutsche Parkinson Gesellschaft (German Parkinson Society)” were used. All experts participated voluntarily and were unpaid, but they had the opportunity to participate in a lottery of three vouchers at 50 € for an internet shop. Experts were informed of study purpose and assessment procedures by email before receiving the link to the survey.

## 3. Results

### 3.1. Study Participants

For the first Delphi round, 145 experts were invited to participate. The survey was completed by 21% (*n* = 30/145) of the initial contacted experts. These 30 experts were invited for the second Delphi round. In the second round, 73% (*n* = 22/30) finished the survey. Overall, 15% (*n* = 22/145) completed the study.

The majority of participants was male (63.3%), had a mean age of 40.2 years (range 27–65 years), and worked as consultants (73.3%). Participants’ average work experience was 13.2 years, and a fifth was specifically trained in palliative care (see Table 1).

### 3.2. Pre-Test

According to the neurologists’ initial feedback, (i) the number of items was reduced from 24 to 19, (ii) the categories ‘neuropsychological symptoms’ and ‘changes in personality’ were merged, and (iii) the wording was simplified in a more patient-friendly way (see Table 2). All original items are displayed in the Appendix (Table A1).

### 3.3. Consensus on Recommendations

#### 3.3.1. Delphi Round 1

In summary, 10 out of 19 (52.6%) initially presented recommendations achieved consensus in both assessment criteria. The Delphi panelists did not agree on 9 (47.4%) aspects. These aspects were adjusted based on the text comments for the second assessment (see Table 2).

In the 1st domain ‘timing of integrating recommendations in ADs’ recommendations R-1.2, R-1.3, R-1.5 and R-1.6 achieved consensus in both evaluation criteria (wording and relevance), whereas recommendations on ‘informing affected patients about disease-specific aspects for their ADs’ (R-1.1), and ‘explanation of palliative care options of a drug pump’ (R-1.4) did not. Both reached only 70% agreement in comprehensibility of wording and R-1.4 70% in relevance for clinical practice. Some experts commented on their uncertainty about the actual recipient of the recommendation and suggested to simplify and shorten the wording. All consented and unconsented recommendations of the 1st domain were rephrased to emphasize that the attending physician and not the patient is the recipient of this domains’ recommendations.

In the 2nd domain ‘levodopa carbidopa intestinal gel’ the recommendation on ‘artificial hydration and nutrition with a gastric tube’ (R-2.3) achieved consensus in both criteria (90.0%). Consensus was not reached for the wording and clinical relevance of ‘different therapy options if the patient is unable to take oral medication because of swallowing disorders’ (R-2.1) and for the wording of ‘continuance of the drug pump therapy if the patient is unable to make decision’ (R-2.2). The missing concretisation of medication, patient´s state of consciousness and different therapy options was criticized. Some experts of the Delphi panel suggested formulations giving patients the opportunity to choose either to confirm or to disagree. The two unconsented recommendations were adapted and presented in the second Delphi round.

In the 3rd domain ‘deep brain stimulation’ all recommendations (R-3.1, R-3.2) achieved consensus in both evaluation criteria. Some experts additionally suggested a more precise formulation of the therapy options. The wording was slightly adjusted accordingly (see Table 3).

In the 4th domain ‘swallowing disorder’ consensus was achieved for all recommendations (R-4.1, R-4.2). Some experts advised a stronger differentiation between artificial hydration and nutrition in case of swallowing disorders (R-4.1) and between therapy options for breathing difficulties caused by swallowing disorders (R-4.2). These comments were integrated in formulations of a multiple selection type.

In the 5th domain ‘changes in personality and neuropsychological symptoms,’ the recommendation on ‘determining a person of trust’ (R-5.2) reached consensus in both evaluation criteria (96.7%). The recommendations R-5.1, R-5.3 and R-5.4 were not consented. Some respondents recommended that the decisions in this domain should not be made by patients but by physicians, who are able to take the medical indication of therapies into account before, in a second step, taking the patient´s will into consideration. Some experts pointed out that patients’ ADs might be in conflict with the indicated therapy. The three unconsented recommendations (R-5.1, R-5.3, R-5.4) were dropped from the project and not included into the second Delphi round.

In the 6th domain ‘bladder and rectal problems,’ both recommendations (R-6.1, R-6.2) did not achieve consensus. The decision to ‘change the drug treatment in case of urinary incontinence caused by the medication’ (R-6.1) reached less than 80% agreement in wording (66.7%) and relevance (63.4%). The recommendation was criticized for the potential conflict between the patient´s will and medical indication. Some experts questioned whether patients would be able to understand the clinical significance of the recommendation. The recommendation R-6.1 was dropped from the Delphi study. ‘Different therapy options for obstipation’ (R-6.2) was rated moderately high in relevance for clinical practice (66.7%). Again, experts commented that patients might not understand the recommendation and that this problem only rarely occur in clinical practice. The recommendation R-6.2 was amended accordingly.

#### 3.3.2. Delphi Round 2

After initial analysis, four out of nine unconsented recommendations of the first Delphi round were dropped from the study (see Table 2). The second Delphi round started in July 2019 and the remaining five unconsented recommendations (R-1.1, R-1.4, R-2.1, R-2.2 and R-6.2) of the first Delphi round were presented in a modified version.

Finally, four out of the five recommendations achieved consensus with an agreement of over 80% regarding wording and relevance (R-1.1, R-1.4, R-2.1, R-2.2).

In the 1st domain ‘timing of integrating recommendations in ADs’ for attending physicians, both recommendations (R-1.1, R-1.4) achieved consensus. Some experts suggested to detail the palliative care options of a drug pump (R-1.4).

In the 2nd domain ‘levodopa carbidopa intestinal gel,’ consensus was reached for both recommendations (R-2.1, R-2.2). In the text comments, some experts asked for a more precise explanation of the therapy options in the recommendation on the ‘drug administration with a gastric tube’ (R-2.1). The authors changed this recommendation accordingly.

In the 6th domain ‘bladder and rectal problems,’ the recommendation on ‘therapy options for obstipation’ (R-6.2) could not achieve consensus on the relevance for clinical practice (77.2%). Some experts questioned whether patients are able to reflect the differences of the proposed therapy options of the recommendation and their potential invasiveness. In consequence, the recommendation was dropped.

In a nutshell, 24 initial recommendations were pre-tested, 19 of them evaluated in a first Delphi round of which 10 were consented. Five recommendations were re-evaluated in a second Delphi round and four of them consented, so that in sum a set of 14 recommendations was accepted for the use in ADs of PwP (see Table 3).

## 4. Discussion

Our previous study has shown that the majority of PwP have a well-prepared AD in terms of general end-of-life aspects. However, the AD hardly deals with the possibilities of specific PD therapy [13]. To our knowledge, this is the first study aiming at improving the specificity of ADs of PwP by developing 14 PD-specific recommendations to be integrated into ADs of PwP as well as to be used as instructions for attending physicians. Yet, no international scientific approaches addressed PD-specific amendments for ADs.

We know from the literature that PwP want to be involved in medical decision-making, but are often unfamiliar with the therapeutic options [18]. This is possibly due to the lack of information flow because, on the one hand, patients do not usually turn to neurologists for advice and, on the other hand, only few neurologists counsel PwP concerning their ADs [13]. Patients’ autonomy can partially be maintained through advance care planning [19]. The provision of neurological expertise in form of recommendations can expand the knowledge of the attending physicians and promote awareness for the integration of PD-specific aspects in ADs.

The recommendations deal with typical disease-specific complications [20,21] that should be included in ADs in order to specifically address the desired type and amount of therapy.

In relation to counselling of ADs, the expert panel agreed in assisting PwP by creating an early PD-specific AD with the help of practical examples. This decision is in line with further data of our group indicating the need for information on palliative care of PwP [3]. Kluger et al. also identified the patients’ wish for an early creation of an AD [22]. This is another argument for an early creation of an AD in addition to the fact that the decisional capacity of patients might decline in the course of PD [12].

Recommendations referring to therapy with dopaminergic medication at the end-of-life were classified as relevant by the experts in the Delphi study. Dopaminergic therapy is a major factor for the alleviation of bothersome motor symptoms such as rigor or tremor and discontinuation may even lead to akinetic crises in the end-of-life care of PwP [23]. In case of non-oral advanced therapies, the patient should be informed in advance of the additive palliative care value of an intervention. This information may help the patient to early visualize later secondary advances which is also in line with the patients’ wishes for more information on palliative care [3,24]. In addition to oral administration, the medication can also be administered non-orally via patch, injection or pump. In the case of levodopa carbidopa intestinal gel it can also be used for artificial nutrition [24]. By applying these recommendations, patients can decide in advance whether a drug pump should be part of the therapy if severe swallowing disorders occur. Some of the Delphi experts were concerned about providing too much worrisome information too early to the patient regarding palliative aspects of pump therapy.

Neuropsychiatric symptoms are very frequent in PD and include anxiety, depression, apathy, cognitive decline, hallucinations and disorders of impulse control [3,25,26]. These neuropsychiatric symptoms were rarely addressed in current advance directives of PwP [13] although these symptoms severely reduce patients’ quality of life and decisional capacity [12,26]. Our expert panel agreed that these symptoms should be addressed early in the course of the disease but the latest after their first appearance by the attending physician. The experts considered it necessary to consult a trusted person who could assess the patient’s character in order to evaluate personality changes and neuropsychological symptoms.

Severe swallowing disorders can distract PwP from eating and drinking, impair breathing, and increase the risk of aspiration and pneumonia [27,28]. The presence of these symptoms is often underestimated by patients and physicians and might cause dose-failure of PD medication [28]. Regarding to the relevance in clinical practice the expert panel consented the recommendations for or against feeding tube and tracheostomy as possible invasive rescue therapy for swallowing disorders.

However, drug safety related recommendations were not consented in the panel, highlighting the difficulty of generalizing the complex and individual decisions concerning medication and treatment goals. As many of these patients were in an geriatric age, multimorbid and under polypharmacotherapy [23]. Comments from the panel state that decisions on medication should be in the hands of the attending physician, as they have the necessary specialist knowledge. The experts were hoping not to overburden PwP by medical treatment decisions because these situations are complex and depend on broad pharmacological knowledge [23,29,30].

Another potential complication in the advanced stage of Parkinson’s disease are bladder and rectal disorders. Associated symptoms such as pain, urge or constipation can be safely treated with botulinum toxin However, this therapy and other possible invasive therapies for these symptoms could not find agreement to be integrated in the recommendations. The experts pointed out that the invasive treatment of bladder or rectal disorders is extremely rare in practice and, therefore, not relevant for inclusion in ADs.

### Limitations

The presented results refer to the current status of ADs of PwP in Germany. Only neurologist and palliative care experts from the German health system took part in the study. The recommendations are related to a Middle European cultural background and may not easily be transferable to other cultural contexts. In order to select the most important items for specific medical treatment situations, only physicians were included in this study. In future studies, the integration of other stakeholders (e.g., lawyers, caregivers, patients and patients’ representatives) is essential for additional improvements. Further, these results were designed for the German health care context which might limit the generalizability of the results. However, the major complications in the course of advanced PD patients are addressed by the present recommendations. Before using the recommendations in other countries, we suggest an adaptation based on specific law cases of the country. Collaboration with experts from diverse countries could further help to overcome the limitations in generalizability.

The expert participation rate of 15% could indicate a ‘selection bias’ in data collection. However, apart from the rather low response rate in our study, we were able to include experts from the fields of palliative care and neurology and had a vivid and constructive discussion of the items. The number of participants appears to be sufficiently high for an effective expert panel.

Unfortunately, we were unable to address the point delirium in our panel. This is an important aspect because a relevant proportion of PD patients die in hypoactive delirium ([31], personal communication with S. Lorenzl). However, this aspect could be integrated in a second revised version of the recommendations.

Finally, this work did not include the caregiver perspective and experience at this stage. However, caregivers are important for the daily support of the patient and play an important role in ACP [16]. Further these people are significantly burdened by caregiving for a PwP [32,33,34]. In future projects, feedback from clinical use and results from scientific evaluations of physicians, patients and caregivers will help to optimize these recommendations.

## 5. Conclusions

People without chronic diseases should include statements on general complications in an end-of-life setting in their ADs and receive consultation from primary care services such as general practitioners. If a severe or chronic disease, such as PD, emerges, patients should be counselled by a specialist regarding disease specific aspects for ADs.

The consented recommendations from this study should be considered when creating or adapting ADs of PwP. Professionals should be trained in counselling and in integrating the present recommendations in ADs.

This Delphi panel approach and the resulting recommendations could serve as a model both for other chronic conditions and for other countries where life-long care requires important decisions that should be considered timely in ADs.

## Figures and Tables

**Table 1:** Demographic data of the consensus experts.

Item	Answer Option	*n* = 30
Age (years)	Mean ± SD	40.2 ± 10.4
Sex	Female	11 (36.7%)
	Male	19 (63.3%)
Level of professional education	Resident	8 (26.7%)
	Consultant	22 (73.3%)
Work experience (years)	Mean ± SD	13.2 ± 9.5
Special training in palliative care	Yes	6 (20%)
	No	24 (80%)

Abbreviations: SD standard deviation.

**Table 2:** Central domains and number of Delphi recommendations.

	Domain	Initial Number of Items	Pre-Test		Delphi Round 1			Delphi Round 2		
			Adapted	Dropped Out	Consented	Unconsented		Consented	Unconsented	
						Adapted	Dropped Out		Adapted	Dropped Out
1.	Timing of integrating recommendations in ADs	6	6	0	4	2	0	6	0	0
2.	Levodopa carbidopa intestinal gel	4	3	1	1	2	0	3	0	0
3.	Deep brain stimulation	2	2	0	2	0	0	2	0	0
4.	Swallowing disorders	2	2	0	2	0	0	2	0	0
5.	Changes in personality	4	4	4	1	0	3	1	0	0
6.	Neuropsychological symptoms	4								
7.	Bladder and rectal problems	3	2	1	0	1	1	0	0	1
	Overall	24	19		15			14		

**Table 3:** Level of agreement on wording and relevance for Delphi statements.

Recommendation	Consented in Round	Delphi Statement	Agreement Wording		Agreement Relevance	
			% 1–2	*n*	% 1–2	*n*
		**1st Domain: Timing of integrating recommendations in ADs**				
R-1.1	2	As part of Advance Care Planning, physicians should discuss disease-specific aspects for an existing or future advance directive with patients suffering from Parkinson’s disease.	86.3	18	100	22
R-1.2	1	Medical discussions on Parkinson-specific phrases for advance directives should take place at a point in time when the patient is able to grasp the complexity of the decisions, including the consequences.	93.3	28	96.7	29
R-1.3	1	Physicians should focus on case examples of Parkinson-specific problems to better illustrate the disease specific decisions for advance directives to patients.	96.7	29	100	30
R-1.4	2	Physicians should provide information about the added medical value of palliative care when explaining or installing a medication pump. Added medical value of palliative care may include help with nutrition or administration of fluids.	81.8	18	90.9	20
R-1.5	1	At an advanced stage of the disease, at the latest, physicians should provide information about swallowing disorders and treatment options for genitourinary symptoms and functional disorders of the rectum. Options may include catheters and Botox injections.	90.0	27	90.0	27
R-1.6	1	Physicians should inform about neuropsychiatric symptoms and their treatment early in the course of the disease, but at the latest after primary appearance.	80.0	24	83.3	25
		**2nd Domain: Levodopa carbidopa intestinal gel**				
R-2.1	2	If swallowing disorders occur in the course of my illness, which make it impossible for me to swallow medication, I: (a) would like to receive necessary medication by other means (e.g., via the vein, via a nasogastric tube, PEG feeding tube, as an injection into the skin). (b) I do not want to receive any more medication, even if this can worsen my state of health.	95.5	21	95.5	21
R-2.2	2	Should my decision-making ability be restricted in the course of my illness, I will make a prior decision as to whether or not to take the appropriate action: (a) continuation of the pump-delivered therapy in order to maintain my mobility. (b) termination of the pump-delivered therapy.	95.5	21	95.5	21
R-2.3	1	Should I receive Duodopa for Parkinson’s therapy via a gastric tube during the course of my illness, I would like to use this tube to additionally: (a) be fed. (b) be supplied with liquid.	90.0	27	90.0	27
		**3rd Domain: Deep brain stimulation**				
R-3.1	1	If, in the course of my illness, the deep brain stimulation (DBS) should no longer work according to the assessment of my specialized treating physicians and the battery of the stimulator should be empty, I wish: (a) one/no operational change of the battery. (b) one/no further charging of the battery.	86.7	26	90.0	27
R-3.2	1	If the deep brain stimulation no longer works well in the course of my illness, I would like (no) further charging of the battery if the stimulator battery is empty.	80.0	24	93.3	28
		**4th Domain: Swallowing disorder**				
R-4.1	1	If, in the course of my illness, severe swallowing disorders occur that cannot be treated sufficiently with conventional therapies, I agree to a PEG feeding tube (PEG): (a) for nutrition/I do not agree. (b) for the administration of liquid/I do not agree.	93.3	28	96.7	29
R-4.2	1	If, in the course of my illness, severe swallowing disorders occur, which also affect my breathing and are not sufficiently treatable with conventional therapies, I wish: (a) that my airways are protected by the installation of a tracheostoma. (b) no tracheostoma system. In this case I am aware of the possibility of a deterioration of my state of health. (c) symptom-relieving measures	83.3	25	86.7	26
		**5th Domain: Changes in personality and neuropsychological symptoms**				
R-5.1	1	In order to assess my behavior, mood, perception, communication skills or mental capacity, please contact my next of kin, XXX.	96.7	29	96.7	29

## Appendix A

**Table A1:** Original items.

**1st Domain: Timing of integrating recommendations in ADs**		
1.1.	PwP sollten so früh wie möglich und proaktiv auf die Integrierung spezifischer Aspekte ihrer Erkrankung in eine vorliegende PV oder eine geplante PV aufmerksam gemacht werden.	Patients suffering from Parkinson’s disease should as early as possible and proactively be made aware of the integration of disease-specific aspects into an existing or planned living will.
1.2.	Spezifische Handlungsanweisungen sollten dann beraten und gemeinsam gefunden werden, wenn der Patient noch in der Lage ist komplexe Entscheidungen und deren Konsequenzen zu überblicken.	Specific instructions should be discussed and defined when the patient is still able to understand the complexity of the decisions, including the consequences.
1.3.	Spezifische Szenarien können dabei helfen, dem Patienten die allgemeinen krankheitsspezifischen Entscheidungen zu verdeutlichen.	Specific scenarios can help to clarify the general disease-specific decisions for the patient.
1.4.	Bereits bei der Aufklärung über nicht-orale Folgetherapien sollte auch über palliativmedizinische Aspekte dieser Therapie aufmerksam gemacht werden.	During the education about non-oral therapies, attention should also already be drawn to palliative medical aspects of this therapy.
1.5.	Spätestens in einem fortgeschrittenen Stadium der Erkrankung sollte über Schluckstörungen und invasive Behandlung vegetativer Symptome wie Blasen- und Mastdarmstörungen gesprochen werden.	Swallowing disorders and invasive treatment of vegetative symptoms, such as bladder and rectal disorders, should be discussed in an advanced stage of the disease at the latest.
1.6.	Bereits früh im Verlauf der Erkrankung, aber spätestens nach dem ersten Auftreten dieser Symptome, sollte über neuropsychiatrische Symptome und deren Behandlung im Rahmen palliativer Versorgungssituationen gesprochen werden.	Neuropsychiatric symptoms and their treatment in palliative care situations should be discussed early in the course of the disease, but at the latest after the first appearance of these symptoms.
**2nd Domain: Levodopa carbidopa intestinal gel**		
2.1.	Im Falle schwerer Schluckstörungen, die es mir unmöglich machen Medikamente/Parkinsonmedikamente zu schlucken, möchte ich (nicht) über alternative Applikationswege behandelt werden.	In case of severe swallowing disorders that make it impossible for me to swallow medications/Parkinson’s medications, I would (not) like to be treated via alternative routes of application.
2.2.	Im Falle einer schweren Demenz wünsche ich (nicht), sofern medizinisch indiziert, die Fortführung der LCIG Therapie.	In the case of the occurrence of severe dementia, I (do not) wish the continuation of medically indicated LCIG therapy.
2.3.	Sollte eine LCIG Therapie eingeleitet werden, möchte ich über diese (nicht) ernährt/mit Flüssigkeit versorgt werden.	If a LCIG therapy should be initiated, I would (not) like to be nourished/supplied with liquid via this therapy.
2.4.	Ich wünsche/verweigere die Gabe von Flüssigkeiten und Ernährung über den PEG Schenkel des LCIG Systems im fortgeschrittenen Stadium der Parkinsonerkrankung.	I wish/deny the administration of fluids and nutrition via the PEG leg of the LCIG system in advanced stages of Parkinson’s disease.
**3rd Domain: Deep brain stimulation**		
3.1.	Im Falle einer ausbleibenden Wirkung der Stimulation mit optimalen Parametern, wünsche ich bei Batterieerschöpfung keinen/einen Stimulatorwechsel oder eine Aufladung.	If the stimulation with optimal parameters is not effective, I do not wish to change or recharge the stimulator when the battery is exhausted.
3.2.	Sollte ich selber oder Dritte Zeichen von Suizidalität unter THS bei mir bemerken, bitte ich um sofortige Anpassung meiner Therapie.	Should I or third parties notice signs of suicidal tendencies under THS, my therapy should be adjusted immediately.
**4th Domain: Swallowing disorder**		
4.1.	Sollten im Verlauf meiner Erkrankung schwere Schluckstörungen auftreten, die mit konservativen Therapien nicht behandelbar sind, stimme ich einer PEG-Anlage zur Sicherung der Atemwege zu/nicht zu.	If severe swallowing disorders occur in the course of my illness, which cannot be treated with conservative therapies, I agree/disagree with a PEG-plant for securing the airways.
4.2.	Sollten im Verlauf meiner Erkrankung schwere Schluckstörungen auftreten, die mit konservativen Therapien nicht behandelbar sind, wünsche ich eine/keine symptomatische Therapie einer möglichen Atemnot notfalls auch mit einer Tracheostoma-Anlage.	If severe swallowing disorders occur in the course of my illness, which cannot be treated with conservative therapies, I would (not) like to have a symptomatic therapy for possible respiratory distress, if necessary, also with a tracheostoma system.
**5th Domain: Changes in personality and neuropsychological symptoms**		
5.1.	Sollten meine Parkinsontherapie meine Verhaltensweisen und Kommunikations-fähigkeit einschränken bzw. verändern, wünsche ich eine/keine umgehende Anpassung bzw. Beendigung dieser Therapien.	If my Parkinson’s therapy limits or changes my behaviour and communication skills, I (do not) wish immediate adaption or termination of these therapies.
5.2.	Für die Identifikation von Veränderungen bzw. Einschränkungen meiner Verhaltensweisen und Kommunikations-fähigkeit soll bitte Rücksprache mit XXX als Person meines Vertrauens gehalten werden.	For the identification of changes or limitations in my behaviour and communication skills, please consult XXX as a person of my trust.
5.3.	Sollten eingesetzte Medikamente einen negativen Einfluss auf meine kognitiven Fähigkeiten haben, bitte ich um eine/keine sofortige Anpassung meiner Therapie. Eine Anpassung der Therapie kann zu einer Einschränkung der motorischen Funktionen führen.	If any of the medications used have a negative influence on my cognitive abilities, I (do not) request immediate adjustment of my therapy. An adjustment of the therapy can lead to a restriction of motor functions.
5.4.	Sollte bei meinen behandelnden Ärzten Unsicherheit über das Vorliegen von neuropsychologischen Störungen im Rahmen der Parkinsonerkrankung bestehen, dann wünsche ich eine/keine Konsultation eines spezialisierten Neurologen zur Diagnostik und Anpassung meiner Therapie.	If my treating physicians are uncertain about the presence of neuropsychological disorders in the context of Parkinson’s disease, I would (not) like to have a consultation with a specialized neurologist for diagnosis and adjustment of my therapy.
**6th Domain: Bladder and rectal problems**		
6.1.	Sollte ich im Verlauf meiner Erkrankung eine schwerwiegende Dranginkontinenz entwickeln, welche unter konservativer Therapie für mich subjektiv nicht ausreichend gelindert werden kann, dann wünsche ich (nicht) eine Katheterversorgung.	Should I develop severe urge incontinence during the course of my illness which cannot be sufficiently relieved under conservative therapy according to my subjective assessment, I (do not) want a catheter supply.
6.2.	Sollten sich meine kognitiven Fähigkeiten durch die medikamentöse Therapie meiner Dranginkontinenz verschlechtern, wünsche ich (nicht) das zeitnahe Absetzen dieser Medikamente.	Should my cognitive abilities deteriorate as a result of the drug therapy of my urge incontinence, I do (not) wish to discontinue these drugs in the near future.

## References

[1] Heinzel S., Berg D., Binder S., Ebersbach G., Hickstein L., Herbst H., Schmedt N. (2018). Do We Need to Rethink the Epidemiology and Healthcare Utilization of Parkinson’s Disease in Germany?. Front. Neurol..

[2] Tuck K.K., Zive D.M., Schmidt T.A., Carter J., Nutt J., Fromme E.K. (2015). Life-sustaining treatment orders, location of death and co-morbid conditions in decedents with Parkinson’s disease. Parkinsonism Relat. Disord..

[3] Klietz M., Tulke A., Muschen L.H., Paracka L., Schrader C., Dressler D.W., Wegner F. (2018). Impaired Quality of Life and Need for Palliative Care in a German Cohort of Advanced Parkinson’s Disease Patients. Front. Neurol..

[4] Fall P.A., Saleh A., Fredrickson M., Olsson J.E., Granerus A.K. (2003). Survival time, mortality, and cause of death in elderly patients with Parkinson’s disease: A 9-year follow-up. Mov. Disord..

[5] Macleod A.D., Taylor K.S., Counsell C.E. (2014). Mortality in Parkinson’s disease: A systematic review and meta-analysis. Mov. Disord..

[6] Bugalho P., Ladeira F., Barbosa R., Marto J.P., Borbinha C., Salavisa M., Conceição Ld Saraiva M., Fernandes M., Meira B. (2019). Motor and non-motor function predictors of mortality in Parkinson’s disease. J. Neural Transm..

[7] Miyasaki J., Kluger B. (2015). Palliative care for Parkinson’s disease: Has the time come?. Curr. Neurol. Neurosci. Rep..

[8] Lum H.D., Sudore R.L., Bekelman D.B. (2015). Advance care planning in the elderly. Med. Clin. N. Am..

[9] Sudore R.L., Lum H.D., You J.J., Hanson L.C., Meier D.E., Pantilat S.Z., Matlock D.D., Rietjens J.A.C., Korfage I.J., Ritchie C.S. (2017). Defining Advance Care Planning for Adults: A Consensus Definition From a Multidisciplinary Delphi Panel. J. Pain Symptom Manag..

[10] Brinkman-Stoppelenburg A., Rietjens J.A., van der Heide A. (2014). The effects of advance care planning on end-of-life care: A systematic review. Palliat. Med..

[11] Houben C.H.M., Spruit M.A., Groenen M.T.J., Wouters E.F.M., Janssen D.J.A. (2014). Efficacy of advance care planning: A systematic review and meta-analysis. J. Am. Med. Dir. Assoc..

[12] Abu Snineh M., Camicioli R., Miyasaki J.M. (2017). Decisional capacity for advanced care directives in Parkinson’s disease with cognitive concerns. Parkinsonism Relat. Disord..

[13] Klietz M., Öcalan Ö., Schneider N., Dressler D., Stiel S., Wegner F. (2019). Advance Directives of German People with Parkinson’s Disease Are Unspecific in regard to Typical Complications. Parkinson’s Dis..

[14] Connolly S., Galvin M., Hardiman O. (2015). End-of-life management in patients with amyotrophic lateral sclerosis. Lancet Neurol..

[15] Lum H.D., Jordan S.R., Brungardt A., Ayele R., Katz M., Miyasaki J.M., Hall A., Jones J., Kluger B. (2019). Framing advance care planning in Parkinson disease: Patient and care partner perspectives. Neurology.

[16] Hsu C.-C.S., Brian A. (2007). The Delphi Technique: Making Sense of Consensus. Pract. Assess. Res. Eval..

[17] von der Gracht H.A. (2012). Consensus measurement in Delphi studies. Review and implications for future quality assurance. Technol. Forecast. Soc. Chang..

[18] Nijhuis F.A.P., van den Heuvel L., Bloem B.R., Post B., Meinders M.J. (2019). The Patient’s Perspective on Shared Decision-Making in Advanced Parkinson’s Disease: A Cross-Sectional Survey Study. Front. Neurol..

[19] Robinson M.T., Holloway R.G. (2017). Palliative Care in Neurology. Mayo Clin. Proc..

[20] Eva Weck C., Lorenzl S. (2018). Neuropalliative care aspects in patients with Parkinson’s disease. Fortschr. Neurol. Psychiatr..

[21] Martinez-Martin P., Schapira A.H., Stocchi F., Sethi K., Odin P., MacPhee G., Brown R.G., Naidu Y., Clayton L., Abe K. (2007). Prevalence of nonmotor symptoms in Parkinson’s disease in an international setting; study using nonmotor symptoms questionnaire in 545 patients. Mov. Disord..

[22] Kluger B.M., Shattuck J., Berk J., Sebring K., Jones W., Brunetti F., Bekelman D.B. (2019). Defining Palliative Care Needs in Parkinson’s Disease. Mov. Disord. Clin. Pract..

[23] Klietz M., Greten S., Wegner F., Hoglinger G.U. (2019). Safety and Tolerability of Pharmacotherapies for Parkinson’s Disease in Geriatric Patients. Drugs Aging.

[24] Lex K., Kundt F., Lorenzl S. (2018). Using tube feeding and levodopa-carbidopa intestinal gel application in advanced Parkinson’s disease. Br. J. Nurs..

[25] Buter T.C., van den Hout A., Matthews F.E., Larsen J.P., Brayne C., Aarsland D. (2008). Dementia and survival in Parkinson disease: A 12-year population study. Neurology.

[26] Balestrino R., Martinez-Martin P. (2017). Neuropsychiatric symptoms, behavioural disorders, and quality of life in Parkinson’s disease. J. Neurol. Sci..

[27] Umemoto G., Furuya H. (2019). Management of Dysphagia in Patients with Parkinson’s Disease and Related Disorders. Intern. Med..

[28] Buhmann C., Bihler M., Emich K., Hidding U., Potter-Nerger M., Gerloff C., Niessen A., Flugel T., Koseki J.C., Nienstedt J.C. (2019). Pill swallowing in Parkinson’s disease: A prospective study based on flexible endoscopic evaluation of swallowing. Parkinsonism Relat. Disord..

[29] Lingor P., Csoti I., Koschel J., Schrader C., Winkler C., Wolz M., Reichmann H. (2016). The Geriatric Patient with Parkinson’s Disease-A Neurological Challenge. Fortschr. Neurol. Psychiatr..

[30] Müller-Rebstein S., Trenkwalder C., Ebentheuer J., Oertel W., Culmsee C., Höglinger G. (2017). Drug Safety Analysis in a Real-Life Cohort of Parkinson’s Disease Patients with Polypharmacy. CNS Drugs.

[31] Ebersbach G., Ip C.W., Klebe S., Koschel J., Lorenzl S., Schrader C., Winkler C., Franke C. (2019). Management of delirium in Parkinson’s disease. J. Neural. Transm..

[32] Klietz M., Rippena L., Lange F., Tulke A., Paracka L., Dressler D., Wegner F. (2019). Validating the Parkinson’s disease caregiver burden questionnaire (PDCB) in German caregivers of advanced Parkinson’s disease patients. Int. Psychogeriatr..

[33] Schmotz C., Richinger C., Lorenzl S. (2017). High Burden and Depression Among Late-Stage Idiopathic Parkinson Disease and Progressive Supranuclear Palsy Caregivers. J. Geriatr. Psychiatry Neurol..

[34] Mosley P.E., Moodie R., Dissanayaka N. (2017). Caregiver Burden in Parkinson Disease: A Critical Review of Recent Literature. J. Geriatr. Psychiatry Neurol..

